# Tensile properties of glaucomatous human sclera, optic nerve, and optic nerve sheath

**DOI:** 10.1007/s10237-024-01872-0

**Published:** 2024-08-08

**Authors:** Joseph Park, Immi Lee, Somaye Jafari, Joseph L. Demer

**Affiliations:** 1 Department of Ophthalmology and Stein Eye Institute, University of California, Los Angeles, 100 Stein Plaza, Los Angeles, CA 90095-7002 USA; 2 Department of Integrative Biology and Physiology, University of California, Los Angeles, Los Angeles, CA USA; 3 Department of Neurology, University of California, Los Angeles, Los Angeles, CA USA; 4 Neuroscience Interdepartmental Program, University of California, Los Angeles, Los Angeles, CA USA; 5 Bioengineering Department, University of California, Los Angeles, Los Angeles, CA USA

**Keywords:** Glaucoma, Optic nerve, Optic nerve sheath, Sclera, Tensile

## Abstract

We characterized the tensile behavior of sclera, optic nerve (ON), and ON sheath in eyes from donors with glaucoma, for comparison with published data without glaucoma. Twelve freshly harvested eyes were obtained from donors with history of glaucoma, of average age 86 ± 7 (standard deviation) years. Rectangular samples were taken from anterior, equatorial, posterior, and peripapillary sclera, and ON sheath, while ON was in native form and measured using calipers. Under physiological temperature and humidity, tissues were preconditioned at 5% strain before loading at 0.1 mm/s. Force–displacement data were converted into engineering stress–strain curves fit by reduced polynomial hyperelastic models and analyzed by tangent moduli at 3% and 7% strain. Data were compared with an age-matched sample of 7 published control eyes. Optic atrophy was supported by significant reduction in ON cross section to 73% of normal in glaucomatous eyes. Glaucomatous was significantly stiffer than control in equatorial and peripapillary regions (*P* < 0.001). However, glaucomatous ON and sheath were significantly less stiff than control, particularly at low strain (*P* < 0.001). Hyperelastic models were well fit to stress–strain data (*R*^2^ > 0.997). Tangent moduli had variability similar to control in most regions, but was abnormally large in peripapillary sclera. Tensile properties were varied independently among various regions of the same eyes. Glaucomatous sclera is abnormally stiff, but the ON and sheath are abnormally compliant. These abnormalities correspond to properties predicted by finite element analysis to transfer potentially pathologic stress to the vulnerable disk and lamina cribrosa region during adduction eye movement.

## Introduction

The biomechanical behaviors of ocular tissues are broadly important to the understanding of normal ocular development and function, and to the pathogenesis and treatment of ocular diseases. For example, the elastic properties of the sclera are influential upon ocular size and refractive error (Metlapally and Wildsoet 2015). The local mechanical properties of the eye are important to understanding ocular trauma (Lam et al. 2022), needle injection (Matthews et al. 2014), and to ocular surgery (Shin et al. 2013). Biomechanical properties of the extraocular muscles are important to understanding their normal function (Shin et al. 2012, 2015; Yoo et al. 2009, 2011), and response to surgery or injury (Laursen and Demer 2011). The biomechanical properties of tissues in and around the optic disk are important to understanding its pathologic responses to elevated intracranial pressure (Sibony 2016) and microgravity (Kesserwani 2021; Raykin et al. 2017; Reilly et al. 2023).

Hydrostatic pressure has been regarded as particularly important to the eye. Intraocular pressure (IOP) maintains ocular shape, but imposes stresses and strains on the sclera and optic disk. Abnormally elevated IOP is damaging in congenital and juvenile glaucoma (Chang et al. 2017a), as well as in secondary glaucomas such as angle closure (Zhang et al. 2017), uveitic (Tan et al. 2018), and traumatic (Bai et al. 2009) glaucoma. However, since many patients with primary open angle glaucoma lack abnormally elevated IOP (Ha et al. 2019; Iwase et al. 2004; Kim et al. 2011; Shi et al. 2013; Zhao et al. 2019), other sources of ON stress are probably important causes of damage. For example, mechanical perturbation to the eye arises from its normal rotational movement. As the eye rotates, the ON and its sheath mechanically load the eye, deforming tissues in and around the optic disk, tethering the eye in large angle adduction (Demer 2016), and sometimes causing the eye to retract (Demer 2018). Deformations of the optic disk and Bruch’s membrane produced by eye movements are much larger than those resulting from very high IOP (Wang et al. 2015), specifically in people who have normal pressure glaucoma (Chuangsuwanich et al. 2023). Since ON length is short relative to the length of the eye socket, tethering of the ON in large angle adduction is particularly important for ocular loading (Clark et al. 2021; Demer et al. 2020; Jafari et al. 2021; Le et al. 2020a; Lim et al. 2023; Park et al. 2023b, 2022; Suh et al. 2018, 2017).

A powerful approach to understanding the role of biomechanics in ON disease is finite element modeling (FEM). Such models represent tissues or organs as meshes of 3-dimensional elements having individually defined mechanical properties, such as hyperelasticity or viscoelasticity, and subject both to mutual interactions at their interfaces, as well as to external boundary conditions and forcing functions. The FEM technique has been applied to simulate effects of IOP elevation and horizontal eye rotation. However, the numerical accuracy of FEM, and thus the conclusions that may be derived from it, may be more sensitive to the accuracy of tissue material properties (Sigal et al. 2009) than to variations in mopre readily measurable tissue geometry (Sigal and Ethier 2009). We previously characterized, in form suitable for FEM, the hyperelastic tensile properties of various regions of human sclera, ON, and ON sheath, derived from testing of fresh eye bank donations without history of glaucoma (Park et al. 2021). Our prior FEM investigation simulating the stresses and strains in the ON and posterior eye caused by ON tethering in large angle adduction suggested that certain combinations of local tissue material properties in the observed range might eventually become pathological. The current study characterizes the ocular tissue properties in eyes with history of glaucoma, allowing direct comparison with FEM predictions of the effects of adduction tethering.

## Methods

### Specimens

We studied 12 human eyes with attached ONs whose donors had been diagnosed with glaucoma before death, but without information concerning the type of glaucoma or relative severity in the two eyes. Specimens were fresh and unfixed, collected within 3 days of death by the Lions Gift of Sight Eye Bank, Saint Paul, MN. Donors were primarily Caucasian, equally representing males and females, of mean age of 86 ± 7 years old (mean ± standard deviation, SD, range 74–95 years). In one eye, the ON was too short for tensile testing due to technique employed by the eye bank harvester. Control specimen numbers varied in the prior study due to variations in harvesting technique and occasional technical failures during testing.

As a control group, we selected data from our prior study those specimens tested with preconditioning identical to the current study (Park et al. 2021), and that were at least as old as the youngest donor in the group with glaucoma, 74 years. The same investigator J.P. performed the experiments for both the glaucomatous and control specimens, using the same apparatus, and the same shipping and preparation methods, including timing. The mean age of the resulting 7 donors (14 eyes) comprising the control group was therefore 84 ± 8 years (range 74–101 years), similar to the mean age of the glaucomatous tissue donors. Both eyes of each donor were tested. For a robust comparison with a large sample, diameter of the healthy ON was the average of digital caliper measurements in 47 fresh eyebank eyes without history of glaucoma that underwent testing in the prior study, including all later tested with and without preconditioning (Park et al. 2021).

### Preparation

**Fig. 1:**
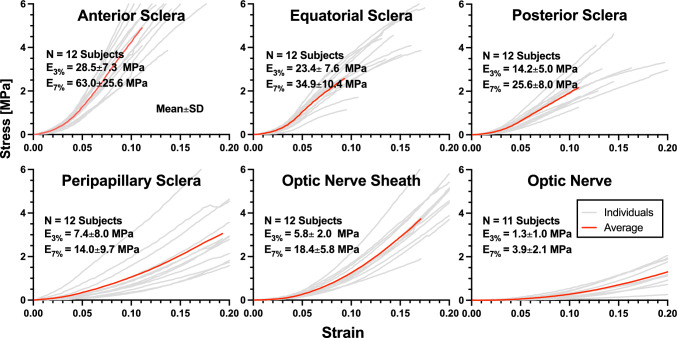
Stress–strain curves of human ocular tissues with glaucoma. Red solid curves indicate mean behaviors up to failure strain of the first specimen, and light gray lines represent individual specimens. Data are truncated to 0.20 strain for graphical clarity. E_3%_ and E_7%_ represent tangent moduli at lower (3%) and higher (7%) strain, respectively. *N* number of subjects (eyes). Error values are standard deviations

Experimental preparation steps for scleral tissue dissection, peripapillary sclera extraction, and ON and its sheath preparation are identical to a previously designed experiment done with eyes free of ocular diseases (Park et al. 2021), so are only summarized here. Extraocular muscles and connective tissues remaining on the eyes were removed, and the sclera was dissected and carefully trimmed. During preparation, eyes were stored in ice cold-lactated Ringer’s solution. Tissues were divided into six regions: anterior, equatorial, posterior, and peripapillary sclera, ON, and ON sheath, according to the diagram in Fig. 1A of our report on the control specimens (Park et al. 2021). Specimens were prepared in rectangular shape, except for ON that was tested in native cylindrical shape. For anterior, equatorial, and posterior sclera, four specimens from each eye were oriented with longer dimension meridionally and four circumferentially. Two specimens of peripapillary sclera from each eye were tested, both oriented circumferentially to the optic disk. For each ON sheath, two specimens were oriented with longer dimension longitudinally and two circumferentially. The ends specimens were secured to thin cardboard tabs using cyanoacrylate to avoid slippage on tensile clamps.

Scleral dimensions (length × width ± SD) were measured by a digital caliper and averaged: 5.98 ± 0.20 mm × 1.88 ± 0.36 mm for anterior sclera, 6.02 ± 0.09 mm × 2.03 ± 0.36 mm for equatorial sclera, 5.88 ± 0.36 mm × 2.01 ± 0.32 mm for posterior sclera, and 3.44 ± 0.66 mm × 1.98 ± 0.45 mm for peripapillary sclera. Mean aspect ratios were 3.18, 2.97, 2.93, and 1.74 respectively. The ON sheath had a mean length of 7.21 ± 1.36 mm, but its width varies in different locations throughout orbit (Vaiman et al. 2015). Therefore, the cross-sectional area (area ± SD) of the ON sheath was determined using optical coherence tomography (OCT, Thorlabs Inc., Newton, NJ) after applying 0.05 N of preloading to eliminate slack and was found to be 1.78 ± 0.60 mm^2^. The entire donated ON was tested, and averaged 11.43 ± 2.82 mm length and 3.33 ± 0.35 mm diameter.

### Tensile loading

The tissues were placed into a closed uniaxial load cell as described previously (Park et al. 2021; Shin et al. 2018, 2012). During the experiment, a heated water bath was placed under the specimen to maintain nearly 100% humidity and about 37ºC temperature in the chamber as measured with a thermocouple. Before testing, all specimens were preloaded to 0.05 N to eliminate slack. Our previous study showed that stress–strain curves for postmortem human ocular tissues converge to repeatable responses after approximately 2–3 cycles of preconditioning (Park et al. 2021). All the specimens were therefore preconditioned by 5 cyclic loadings with a maximum strain of 5%. For subsequent tensile testing under displacement control, a constant 0.1 mm/s loading rate was then applied to failure, and strain was measured based on the edge-to-edge distance between specimen clamps.

### Control comparison

Data were compared with that previously published without history of glaucoma (Park et al. 2021), but only including donors at least as old as the youngest specimen in the current series to make the resulting age distribution comparable to the glaucomatous specimens. Comparisons of complete stress–strain curves for each tissue were made, as well as tangent moduli computed for 3% and 7% strains. Tangent moduli were computed by averaging the loading curves for each tissue region in each specimen. Tangent moduli at 3% strain were computed as linear regression slopes for 8 data points incremented by 0.2% from 2.3 to 3.7% strain. Tangent moduli at 7% strain were analogously computed from 6.3% to 7.7% strain.

### Material property analysis

The sclera, optic nerve (ON), and optic nerve sheath (ONS) are modeled as hyperelastic, homogeneous, isotropic, and incompressible materials exhibiting nonlinear behavior. As previously described (Park et al. 2021), engineering stress–strain data were fitted to a Reduced Polynomial hyperelastic model using Abaqus 2020 (Dassault Systèmes SIMULIA Corp., Johnston, RI). Among the Mooney-Rivlin, Ogden (1st-4th order), and reduced polynomial models, the 2nd-order reduced polynomial model was identified as the lowest order and most stable for accurately fitting the experimental stress–strain curves. To minimize noise in future Abaqus simulations, we assumed Poisson’s ratio to be 0.49, consistent with previous assumptions for eye tissues (Muñoz Sarmiento et al. 2023; Wang et al. 2017). The strain energy density function $U$ of the reduced polynomial model is expressed by

1 $$ U = \mathop \sum \limits_{i = 1}^N C_{i0} (\overline{I}_1 - 3)^i + \mathop \sum \limits_{i = 1}^N \frac{1}{D_i }(J_{el} - 1)^{2i} $$

where $$\overline{I}_1$$ is the first invariant of the right Cauchy–Green strain tensor, $$C_{i0}$$ and $$ D_i$$ are material constants, $$J_{el}$$ determines the elastic volume ratio, and $$N$$ is the polynomial order.

### Statistics

Coefficients of variation (CV) were computed as standard deviations divided by means. Two types of statistical tests were performed for comparisons of continuous data. Since this was an exploratory analysis, means of tangent moduli at arbitrary strain levels were compared using Student’s t-tests without adjustment for multiple comparisons. In order to account for possible correlations between the right and left eyes of individual donors, comparisons of complete stress–strain curves for each tissue were made of the method of generalized estimating equations (GEE) implemented in IBM SPSS Statistics 25 (IBM, Armonk, NY, USA), and incorporating donor age as a covariate. This approach accounts for possible correlations between the two eyes of the same subjects and has type 1 error characteristics advantageous over t-testing (Huang et al. 2018). Being limited to three decimal places, it is a feature of GEE to report P values of 0.000 when less than 0.001. Correlations of tensile properties of local tissue regions within the same eyes were evaluated by the mutual correlation matrix for Pearson’s r statistic; values of 0.5 or more are considered strong, 0.3–0.5 moderate, and less than 0.3 weak.

## Results

### Optic nerve diameter

Size of the ON was evaluated as an indicator of glaucomatous optic neuropathy. Mean ON diameter was 3.30 ± 0.34 mm in specimens with history of glaucoma, significantly smaller than the average of 3.86 ± 0.37 mm in control specimens without history of glaucoma that included a total of 47 specimens (*P* < 10^–5^)(Park et al. 2021). The ON cross section of specimens with glaucoma was thus 73% that of the control specimens, consistent with glaucomatous optic atrophy.

### Linear approximation of tensile moduli of ocular tissue with glaucoma

Stress–strain data for glaucomatous ocular tissues are shown Fig. 1. Each curve terminated at the ultimate stress at which failure occurred, and average curves for all experiments are illustrated up to the least ultimate strain among samples of the same tissue.

For anterior sclera, the tangent modulus at 3% strain was 28.5 ± 7.3 MPa, similar to that of the equatorial sclera at 23.4 ± 7.6 MPa but half of the posterior sclera at 14.2 ± 5.0 MPa. Peripapillary sclera had 7.4 ± 8.0 MPa tangent modulus, and ON sheath had 5.8 ± 2.0 MPa tangent modulus. The ON had the least tangent modulus at 1.3 ± 1.0 MPa.

Tangent moduli at a high 7% strain differ slightly among specimens (Fig. 1). For anterior sclera, the 63.0 ± 25.6 MPa tangent modulus at 7% strain was more than twice the 28.5 ± 7.3 MPa value at 3%, and was much larger than the tangent moduli of the equatorial and posterior sclera (Fig. 1). Tangent moduli for equatorial, posterior, and peripapillary sclera were lower that anterior sclera, but also increased (stiffened) with strain (Fig. 1).

### Comparison between glaucomatous and control eyes

**Fig. 2:**
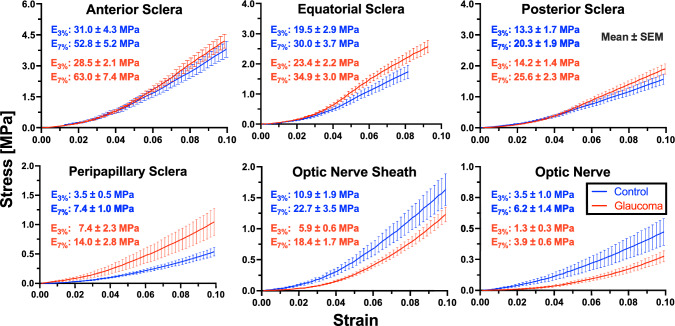
Mean stress–strain behavior comparing eyes with history of glaucoma, versus control eyes. SEM- standard error of mean. All curves for eyes with glaucoma differed significantly from control by generalized estimating equation analysis with a model also including donor age (*P* < 0.001, except for optic nerve sheath, *P* = 0.006, Table 2). *SEM* standard error of the mean

Data from donors with glaucoma were compared to control data from tissues without history of glaucoma obtained in our previous study using identical methods that included preconditioning (Park et al. 2021). Figure 2 compares average stress–strain curves between glaucomatous and healthy tissues for each anatomical region, with tangent moduli ± standard errors of the means. For the anterior sclera, there was a low tow region up to 4% strain in which glaucomatous and control sclera behaved similarly, above which glaucomatous sclera was stiffer than control. This was also the case up to 2% strain for equatorial sclera, and 4% strain for posterior sclera (Fig. 2). However, glaucomatous peripapillary scleral exhibited almost no low toe region, although one was evident up to 1.5% strain for control eyes. The peripapillary sclera of glaucomatous specimens was stiffer than controls throughout the entire range of strain, with tangent moduli almost twice as great at both 4% and 7% strain.

For the ON and its sheath, the trend in tissue behavior was opposite that of the sclera. Throughout the entire range of strain, glaucomatous ON and sheath were less stiff than control tissue (Fig. 2). At 3% strain, the tangent modulus for the glaucomatous ON sheath was 5.9 ± 0.6 MPa, compared with 10.9 ± 1.8 MPa for control. At 7% strain, the tangent modulus for ON sheath was 18.4 ± 1.7 MPa, compared with 22.7 ± 3.5 MPa for control. At 3% strain, the tangent modulus for the glaucomatous ON was 1.3 ± 0.3 MPa, compared with 3.5 ± 1.0 MPa for control. At 7% strain, the tangent modulus for the glaucomatous ON was 3.9 ± 0.6 MPa, compared with 6.2 ± 1.4 MPa for controls.

Statistical comparisons of tangent moduli between glaucomatous and control tissues were performed using t-tests. As may be seen in Table 1, the tangent moduli were significantly different between donor groups only for stiffer peripapillary sclera in glaucoma at 7% strain (*P* = 0.043), and at 3% strain for more compliant ON sheath (*P* = 0.033). While there was a trend for lower tangent moduli for the glaucomatous ON, fewer control specimens were available for comparison, and the difference in glaucoma did not reach significance (*P* = 0.063). In addition, GEE was used to statistically compare all values in the right with left eyes of the two donor groups, but there were no significant differences between the right and left eyes in any tissue areas in either group (*P* > 0.09).

**Table 1:** Tangent moduli of ocular tissues

Regions		Donors	Number of specimens	Tangent modulus at 3% strain (MPa)				Tangent modulus at 7% strain (MPa)			
			N	Mean	SD	CV	*P* value	Mean	SD	CV	*P* value
Sclera	Anterior	Control	9	31.0	12.8	0.41	0.598	52.8	15.7	0.30	0.273
		Glaucoma	12	28.5	7.3	0.26		63.0	25.6	0.41	
	Equatorial	Control	10	19.5	9.0	0.46	0.294	30.0	11.8	0.39	0.317
		Glaucoma	12	23.4	7.6	0.32		34.9	10.4	0.30	
	Posterior	Control	10	13.3	5.3	0.40	0.670	20.3	6.1	0.30	0.097
		Glaucoma	12	14.2	5.0	0.35		25.6	8.0	0.31	
	Peripapillary	Control	10	3.5	1.4	0.40	0.130	7.4	3.3	0.44	0.043
		Glaucoma	12	7.4	8.0	1.09		14.0	9.7	0.69	
Optic nerve sheath		Control	8	10.9	5.3	0.49	0.033	22.7	10.0	0.44	0.291
		Glaucoma	12	5.9	2.0	0.33		18.4	5.8	0.32	
Optic nerve		Control	6	3.5	2.3	0.66	0.063	6.2	3.5	0.56	0.173
		Glaucoma	11	1.3	1.0	0.77		3.9	2.1	0.54	

*P* values by Student t-test

### Variability

We assessed variability of tensile properties using the CV of tangent modulus, computed as standard deviation divided by the mean (Table 1). Data for control tissue are from the published prior study (Park et al. 2021). In most regions, variability was similar to the prior study of specimens without history of glaucoma. For anterior, equatorial, and posterior sclera, as well as ON and ON sheath, the CV for specimens with glaucoma tended to be similar to or less than control. However, tensile variability was greater in glaucomatous peripapillary sclera than in controls. In peripapillary sclera, the CV at 3% strain was much higher at 1.09 for glaucoma than 0.40 for control; the CV at 7% strain was 0.69 for glaucoma, higher than 0.44 for control.

### Complete tensile curve analysis

Tangent moduli capture only linear approximations to slope at specific but arbitrary levels of strain. We additionally used GEE to analyze every acquired stress and strain pairing for each tested specimen, representing the complete experimental curves. Analysis of complete (untruncated) stress–strain curves using GEE models with both age and glaucoma diagnosis as covariates, and accounted for correlations between right and left eyes of individual donors, demonstrated that while age was usually a significant factor, differences attributable to glaucoma were also highly significant in every tissue (Table 2).

**Table 2:** Generalized estimating equation probabilities of glaucoma and age effects on tissue stress–strain functions

Regions		Glaucoma versus control (*P* value)	Age as covariate (*P* value)
Sclera	Anterior	0.000	0.010
	Equatorial	< 0.001	0.583
	Posterior	< 0.001	0.008
	Peripapillary	0.000	0.000
Optic nerve sheath		0.006	< 0.001
Optic nerve		0.000	< 0.001

### Regional correlations

**Fig. 3:**
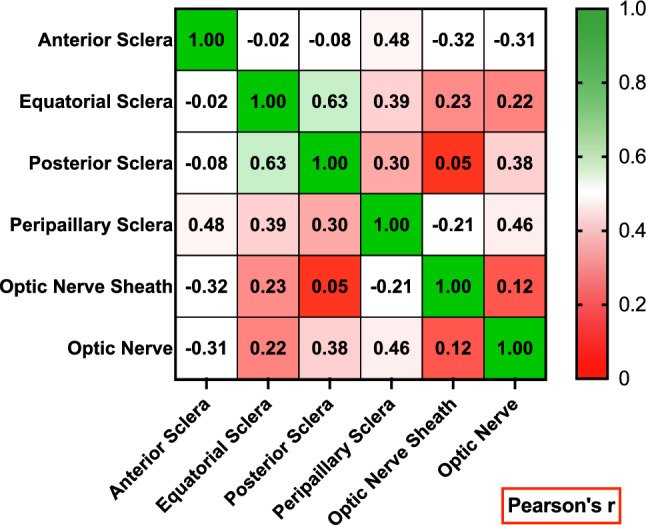
Correlations of tangent moduli at 3% strain among tissue regions for eyes with history of glaucoma. Correlation signs are arbitrary

We considered the possibility that tensile properties of local tissue regions might be correlated within the same eyes. Such correlations were examined for 3% tangent modulus by creating a mutual correlation matrix for Pearson’s r statistic, as shown in Fig. 3. The highest Pearson’s *r* correlation was found between equatorial and peripapillary sclera at 0.63. All other correlations had absolute values less than 0.5. None of these correlations was statistically significant, such that for *r* (11–12), *P* > 0.25. This means that on average, tensile properties in one region of the eye poorly correlate with properties in any other region of the eye.

### Hyperelastic model parameters

Average stress–strain curves of all six ocular regions, which are shown as red solid lines in Fig. 1, were fitted using second order reduced polynomials that are of the lowest order suitable for computational stability as Abaqus simulation package inputs. The reduced polynomial hyperelastic model parameters are shown in Table 3 based on the assumption of Poisson’s ratio, $v$ = 0.49. Coefficients of determination (*R*^2^) for the fits all exceeded 0.98. These models are suitable for FEM, as direct inputs into the software package ABAQUS. As demonstrated in Table 3, Abaqus curve fitting assigns a value of zero to D_2_. Consequently, for *i* = 2, Abaqus disregards the second term of Eq. (1).

**Table 3:** Reduced polynomial hyperelastic parameters of glaucomatous ocular tissues

Regions		C_10_ (MPa)	C_20_ (MPa)	D_1_ (MPa)^−1^	D_2_ (MPa)^−1^
Sclera	Anterior	1.06	149.8	1.90 × 10^–2^	0
	Equatorial	0.62	137.6	3.26 × 10^–2^	0
	Posterior	0.55	68.7	3.67 × 10^–2^	0
	Peripapillary	0.78	15.4	2.59 × 10^–2^	0
Optic nerve sheath		0.24	32.6	8.26 × 10^–2^	0
Optic Nerve		4.23 × 10^–2^	7.5	0.48	0

## Discussion

The present experimental study in postmortem human eyes supported the clinical diagnosis by the objective finding that ON cross section was reduced to only about 73% of control size, consistent with glaucomatous optic neuropathy (Quigley et al. 1982). Tensile characteristics of these eyes with history of glaucoma are consistent with prior characterizations of local regions of human sclera (Chen et al. 2014; Geraghty et al. 2012; Spoerl et al. 2005; Wollensak and Spoerl 2004; Woo et al. 1972), and employ the same reduced polynomial hyperelastic models recently published for human sclera, ON, and its sheath (Park et al. 2021). These polynomial hyperelastic models were chosen to be computationally suitable for FEM using published commercial software (Park et al. 2021), a purpose for which there is no theoretical penalty for possible overdetermination of the fits to the data. The current study demonstrated important relevant tensile differences between glaucomatous and control eyes in tissue regions of potential significance to the pathogenesis of glaucoma.

### Scleral stiffness

Unsurprisingly since rigidity of the anterior sclera is functionally important to stabilize the eye’s optics for clear vision (Curtin 1969), the present study confirms in glaucomatous eyes the previous findings that the anterior sclera is stiffest of all scleral regions (Elsheikh et al. 2010; Friberg and Lace 1988; Geraghty et al. 2012; Park et al. 2021), and only minimally different from control eyes previously reported (Park et al. 2021) (Fig. 2). As in control eyes, scleral stiffness in eyes with glaucoma tended to decrease progressively in more posterior regions, so that peripapillary sclera was the most compliant. As in previous studies, scleral stiffness decreased from anterior to posterior sclera, being least in the peripapillary region (Park et al. 2021). It is notable, however, that equatorial and peripapillary sclera of glaucomatous specimens was greater than control in stress–strain curves (Fig. 2), and that the tangent modulus at 7% strain was significantly greater than control in glaucomatous specimens (Table 1). This finding concords with recent opinion that tissue stiffening is a hallmark of glaucoma, as reviewed (Downs 2015; Liu et al. 2018; Powell et al. 2023) and quantified by Grytz et al. (Grytz et al. 2014) and Fazio et al. (Fazio et al. 2014). However, it has previously been suggested that the scleral changes associated with glaucoma might be results, rather than causes, of the disease (Coudrillier et al. 2012). In glaucoma, the elastic modulus of peripapillary sclera was more variable than normal (Table 1), perhaps reflecting differences in contribution or consequence of disease in individual eyes.

### Optic nerve sheath

The ON sheath is a bilaminar structure that coaxially encircles the ON (Le et al. 2020b; Shin et al. 2020), presumably to protect it from mechanical damage since the elastic modulus of the sheath is at least threefold greater than that of the ON (Fig. 2). The inner layer of the ON sheath is significantly stiffer than the outer layer (Shin et al. 2020). The current study reports the novel demonstration that the glaucomatous ON sheath is significantly less stiff than controls. The tangent modulus at 7% strain of the glaucomatous sheath was only 54% of control (Table 1). This would suggest that the glaucomatous sheath might offer less protection to the ON during eye movements; if so, this deficiency might predispose to the development of glaucoma. Alternatively, however, softening of the ON sheath might instead be a result, rather than cause, of glaucoma.

### Optic nerve

Unlike the glaucomatous sclera that was found here to be as stiff or stiffer than control tissue, the glaucomatous ON was less stiff than normal. It is plausible that optic atrophy resulting from glaucoma could account for lower stiffness of the glaucomatous ON. However, the ON normally contains a significant network of internal connective tissue intimately associated with dense surrounding pia mater that renders the ON much stiffer than any other white matter tract of the central nervous system of which the ON is a part (Garcia and Demer 2023; Karim et al. 2004). Unless glaucomatous atrophy of ON axons also caused at least commensurate connective tissue atrophy, the linear elastic modulus would be expected to increase rather than decrease as a result of axonal loss, since modulus is normalized to tissue cross section. This suggests that decreased ON stiffness might itself be a predisposing factor to glaucoma, although the current data cannot exclude the possibility that decreased ON stiffness might be a result of tissue remodeling induced by glaucoma.

The lower tensile stiffness of the glaucomatous ON reported here would seemingly predict abnormally greater, rather than less, ON extensibility during ON tethering in large adduction. During large adduction, MRI demonstrates that the normal ON elongates by about 0.8 mm (Clark et al. 2021) and tensile strain is distributed uniformly along the length of the ON throughout the orbit, resulting in only about 0.5 mm globe retraction (Lim and Demer 2023). However, MRI performed during incremental adduction from 26° to 32° where ON path is straight demonstrates absence of ON elongation and resultant greater globe retraction in primary open angle glaucoma, regardless of whether there was a history of normal (Clark et al. 2021; Demer et al. 2017) or elevated IOP (Demer et al. 2020). Several factors may contribute to explain this seeming paradox. Since in this study the ON sheath was about threefold stiffer than the ON that it encircles and protects, the tensile load during ON traction in vivo is largely borne by the sheath. Due to stiffening above about 3% strain that affects the ON sheath (Lim et al. 2024), its tangent modulus in glaucomatous specimens approaches that of normal at higher strains, while the modulus of peripapillary sclera remains about twice normal throughout the range of strain (Fig. 2). The peripapillary sclera normally has elastic modulus about one third that of the ON sheath (Fig. 2). In glaucoma by contrast, the peripapillary sclera is stiffer than the ON sheath. This suggests that the absence of globe retraction and presence of ON elongation during adduction tethering in normal subjects is due to a complex interplay of mechanical changes in multiple tissues that could also include the suspensory tissue of the globe, and effects of cerebrospinal fluid within the ON sheath. A quantitative analysis of this interplay would be facilitated by a finite element model of the globe and ON incorporating the extraocular muscles and all relevant supporting tissues. To date, hemisymmetric models incorporating only the horizontal rectus muscles have been developed (Jafari et al. 2024; Park et al. 2022; Shin et al. 2017a; Wang et al. 2017; Wang et al. 2016), but will require considerable computational resources to extend to the degree necessary to clarify all of the mechanical implications of ON loading during eye rotations.

### Effect of age

The present study employed statistical modeling using GEE to demonstrate a significant effect of glaucoma on mechanical properties of the sclera, ON, and ON sheath, independent of a significant effect of age on all of these except for equatorial sclera (Table 2). This result confirms and extends that of Coudrillier and colleagues, who performed posterior eye inflation testing with digital image correlation to measure the biomechanical responses of postmortem human eyes subjected to inflation by elevated IOP (Coudrillier et al. 2012). We concur with Coudrillier et al.’s dual finding that age has a significant effect, but that glaucoma contributes a significant additional effect. However, the present results differ from Coudrillier et al., who did not observe the difference from normal that was observed here in mid-posterior sclera (Coudrillier et al. 2012). We suspect that this may have been the result of boundary conditions on the inflation of only the posterior eye.

### Regional stiffness correlation

While linear tangent moduli incompletely represent hyperelasticity, these linear measures of stiffness are nevertheless useful for statistical correlations among eyes. The correlation matrix in Fig. 3 demonstrates that there is generally low correlation in elastic properties among the various regions of individual glaucomatous eyes, even lower than previously reported for control eyes (Park et al. 2021). The strongest regional correlation of 0.63 was between posterior and equatorial sclera, which indicates only 63% of the variation in posterior scleral tangent modulus is statistically attributable to variation in equatorial scleral modulus. There was low correlation between elastic modulus of anterior sclera, and the moduli of any of the posterior tissues, including ON and its sheath, and peripapillary sclera. This implies that a potentially available in vivo stiffness measurement of the clinically accessible anterior sclera might on average reflect about 48% of the variation in peripapillary scleral stiffness, 32% of ON sheath and 31% of ON stiffness. There was only 12% correlation between elastic moduli of the ON and its sheath, indicating marked discordance in material properties of these anatomically associated tissues. As earlier demonstrated for control eyes (Park et al. 2021), it is not possible accurately to estimate the elastic behavior of one ocular region from a value measured elsewhere in the eye. Conversely, FEM studies of the biomechanics of the eye may reasonably presume that all possible values of local stiffnesses might potentially occur in a single eye. As noted below, some combinations might predispose to optic neuropathy under certain conditions.

### Implications for pathology

Traction exerted by the ON and sheath during eye rotations has recently been recognized as an important mechanical load on the eye (Demer 2016). Optical imaging demonstrates see-saw deformation of the optic disk during even small and moderate ab- and adduction eye movements (Chang et al. 2017b; Sibony 2016; Suh et al. 2017). It has been demonstrated by MRI that ON length is insufficient to avoid tethering the globe when adduction exceeds around 26° (Suh et al. 2017). Further adduction within the approximately 40° oculomotor range requires that the globe also translate, mainly nasally in healthy subjects as the ON itself stretches (Clark et al. 2021), but the globe pathologically retracts in normal tension glaucoma (NTG) (Demer et al. 2017), where the ON does not stretch (Clark et al. 2021). All eye rotations concentrate reaction force against the optic disk, peripapillary retina, & sclera. It has been shown by OCT (Chang et al. 2017b) and scanning laser ophthalmoscopy (Le et al. 2020a; Park et al. 2023a) imaging that adduction tethering locally deforms these visually critical tissues with a strain “fingerprint” closely resembling degeneration patterns typical of glaucoma and axial myopia. Sibony et al. used OCT to show adduction-induced folds extending to the macula in papilledema (Sibony and Hou 2019). Park et al. also demonstrated that adduction causes shearing between the retina and choroid nasal to the optic disk (Park et al. 2023a).

Eye movement-related deformation has been proposed as another mechanical etiology for NTG operating alternatively or in addition to IOP (Demer 2016; Demer et al. 2017; Shin et al. 2017a; Wang et al. 2017). The contribution of adduction has been emphasized, because ON length is insufficient to permit unhindered rotation, tethering the globe in adduction (Demer 2016) greater than about 26° (Suh et al. 2017), but smaller adduction and abduction also significantly deform the eye. While the healthy ON stretches during adduction tethering, in NTG the ON fails to stretch so that it abnormally retracts the globe (Clark et al. 2021) and exaggerates strain on the disk (Chuangsuwanich et al. 2023). Deformations of the disk and Bruch’s membrane produced by eye movements exceed IOP-related deformations suggested as pathological to retina (Fortune 2019), and many-fold those resulting from extreme IOP elevation in angle closure glaucoma (Wang et al. 2015) and NTG (Chuangsuwanich et al. 2023). A recent clue is the population-based observation in Korea that untreated large angle esotropia increases the glaucoma risk sevenfold more than elevated IOP, while exotropia does not (Kim et al. 2020).

Recent FEMs have provided an informative link between eye movements and deformations of the optic disk and peripapillary ocular tissues. These computational models incorporate both structural anatomy, and local material properties of the tissues to the extent they are known, or in the absence of data, assumed. Wang et al. (Wang et al. 2017, 2016) developed an FEM of disk deformations during arbitrarily applied horizontal ductions, but based the FEM on extrapolated tissue properties from a variety of sources and species (Wang et al. 2017, 2016). Shin et al. modeled disk and scleral deformation due to ON tethering in large adduction, but employed measured bovine, rather than human tissue properties, and constrained the globe to rotate arbitrarily about its geometric center (Shin et al. 2017b). Park et al. developed a FEM of ON tethering in large arbitrarily imposed adduction about its center, but more realistically incorporating the human tissue properties measured in our prior study of postmortem human eyes without history of glaucoma (Park et al. 2022). Park et al. also explored using FEM the effect of stress and strain in the ON and posterior sclera of variations in local ocular tissue properties within the range of observed measurements. This FEM sensitivity analysis predicted that the highest stress in the temporal optic disk and ON during adduction tethering would occur when the posterior and peripapillary sclera were highly stiff, but optic nerve sheath was relatively compliant (Case B) (Park et al. 2022). This combination of material properties matches that observed here in ocular specimens with history of glaucoma (Fig. 2), supporting the proposition that this set of material properties may predispose to ON damage from accumulated adductions. The FEM study predicted that the presumably pathological effect of adduction tethering would be increased only slightly by elevated IOP (Park et al. 2022).

### Limitations

Since postmortem donations uncommonly come from people with documented history of glaucoma, this study is understandably limited by sample size even though sample accrual required several years. Only limited clinical information was available regarding the history of glaucoma for the current specimens, and this history did not distinguish laterality or specific forms of glaucoma such as open angle, closed angle, or secondary glaucoma. Specimens were tested up to three days postmortem, but were promptly harvested and then immediately stored in chilled Ringer’s lactate solution; this timing is probably the best that can be achieved with human tissue. The current data consist of uniaxial tensile properties that do not capture potentially anisotropic behavior that might in principle be investigated directly for sclera and ON sheath by biaxial testing, or indirectly by whole globe or ON sheath inflation testing; these alternative approaches have practical limitations for generation of data suitable for FEM. No ex vivo test method can fully replicate the in vivo loading situation.

While we report hyperelastic material properties, coefficients of curve fits are not directly suitable for statistical comparison due to frequent overdetermination by multiple parameters; fits were chosen for computational compatibility with FEM software. Consequently, statistical comparisons of tensile properties between glaucomatous and control eyes were made at arbitrary low and high strain regions (3% and 7%), using calculations that assumed linearly elastic tangent moduli. Of course, this does not imply that we believe that the mechanical properties are actually linear. Nor can the current study determine if or how microstructural changes in glaucomatous tissues due to disease initiation or progression might alter linearity or anisotropy. Nevertheless, the current data provide a starting point for theoretical studies such as FEM of the mechanical effects of pressure and eye movement-related stresses on the human eye in glaucoma.

## Data Availability

Data files available at Zenodo 10.5281/zenodo.12693821.
